# Availability, utilization, and factors associated with professional pharmacy services in community pharmacies in Saudi Arabia: a cross-sectional study

**DOI:** 10.3389/jpps.2026.17413

**Published:** 2026-09-22

**Authors:** Wael Y. Khawagi, Omar N. Al-Sharif, Tariq A. Al-Zahrani, Saeed Saad A. Alghamdi, Wadia S. Alruqayb, Abdullah A. Alshehri

**Affiliations:** 1 Department of Clinical Pharmacy, College of Pharmacy, Taif University, Taif, Saudi Arabia; 2 College of Pharmacy, Taif University, Taif, Saudi Arabia

**Keywords:** community pharmacists, community pharmacy, pharmaceutical care, pharmacy services, Saudi Arabia

## Abstract

**Background:**

Community pharmacists are increasingly expected to provide patient-centered clinical and public health services beyond traditional medication dispensing. However, the availability and utilization of these services in Saudi community pharmacies remain insufficiently characterized.

**Methods:**

A cross-sectional survey was conducted between January and April 2026 among licensed pharmacists working in community pharmacies in Saudi Arabia. A structured online questionnaire assessed the availability and utilization of 26 professional pharmacy services, perceived barriers to service provision, and overall satisfaction. Service availability and utilization scores were calculated, and factors associated with high service utilization were examined using Spearman correlation and multivariable logistic regression.

**Results:**

A total of 188 respondents participated. Core pharmacy services, including prescription dispensing (97.9%) and medication counselling (97.3%), were widely available. In contrast, extended services such as vaccination, health screening, pharmacovigilance, electronic health record access, and private consultation rooms were considerably less available. Higher prescription volume was the strongest independent predictor of high service utilization. The most frequently reported barriers were unclear regulations (55%), workload and time constraints (53%), and administrative burden associated with the Wasfaty system (51%). Overall, 42.0% of respondents reported being satisfied or very satisfied with the current scope of pharmacy services.

**Conclusion:**

Community pharmacists in selected regions of Saudi Arabia reported high availability of core pharmaceutical services, while clinical, preventive, and advanced services were less consistently available. Addressing regulatory, operational, and infrastructure-related barriers and strengthening integration with primary healthcare may enhance the contribution of community pharmacists to healthcare delivery and support broader healthcare transformation goals.

## Introduction

Community pharmacies are among the most accessible healthcare facilities worldwide and represent the first point of contact with the healthcare system for many individuals [1]. Traditionally focused on medication dispensing, the role of community pharmacists has evolved considerably over the past 2 decades toward the delivery of patient-centered pharmaceutical care and public health services [2, 3]. In addition to ensuring the safe and effective use of medicines, community pharmacists now contribute to medication therapy management, chronic disease monitoring, preventive health services, vaccination, health screening, smoking cessation, lifestyle counselling, and medication safety initiatives. This expanded scope has been recognized internationally as an important strategy to improve access to healthcare, optimize medication use, and reduce the burden on primary healthcare services [2, 4–6].

The successful implementation of expanded pharmacy services depends not only on pharmacists’ competencies but also on organizational, regulatory, and operational factors. Previous studies have shown that workforce capacity, professional training, workload, pharmacy infrastructure, reimbursement mechanisms, availability of private consultation areas, and supportive legislation all influence the extent to which pharmacists can deliver clinical and public health services [7–9]. Consequently, the availability of pharmacy services may differ substantially between healthcare systems and even between pharmacies within the same country [1, 10].

Saudi Arabia has undergone substantial healthcare transformation under Vision 2030, with increasing emphasis on strengthening primary healthcare and expanding the contribution of allied health professionals, including pharmacists [11–13]. Community pharmacies have experienced major changes through the introduction of electronic prescribing systems such as Wasfaty, greater integration with digital health platforms, and increasing expectations for pharmacists to participate in patient education, chronic disease management, and preventive healthcare [14–16]. Recent Saudi studies have reported high patient satisfaction with Wasfaty and highlighted the importance of service availability, pharmacist counselling, and system efficiency [17, 18]. These developments have positioned community pharmacists to play a more active role in achieving national healthcare goals, including improving healthcare accessibility and promoting rational medicine use.

Despite these advances, evidence suggests that the implementation of professional pharmacy services in Saudi community pharmacies remains variable. While dispensing and medication counselling are well established, more advanced clinical services, including structured medication review, health screening, vaccination, pharmacovigilance, medication adherence support, and other patient-centered interventions, appear to be inconsistently available [19, 20]. Previous Saudi studies have generally focused on individual services or pharmacists’ perceptions and have often been limited to specific regions or selected aspects of pharmacy practice. National evidence describing the current scope of pharmacy services, their frequency of delivery, and the factors associated with their implementation remains limited [21–25]. Therefore, this study aimed to evaluate the availability and utilization of professional pharmacy services in community pharmacies in Saudi Arabia. In addition, the study sought to identify demographic, professional, and operational factors associated with service provision and to explore pharmacists’ perceived barriers to delivering these services.

## Materials and methods

### Study design and setting

A cross-sectional, descriptive study was conducted between January and April 2026 to evaluate the availability and utilization of professional pharmacy services in community pharmacy settings within Saudi Arabia. The study also aimed to identify the demographic, professional, and operational factors, as well as the perceived barriers, associated with service provision.

### Study population and sampling

The target population comprised licensed pharmacists actively working in community pharmacy settings in Saudi Arabia. Participants were recruited through a non-probability, convenience sampling approach via professional networks, pharmacy associations, and online distribution channels. Inclusion criteria required that respondents be currently employed in a community pharmacy at the time of data collection. Participation was voluntary, and no incentive was offered. A total of 188 respondents completed the questionnaire and were included in the final analysis.

### Data collection instrument

Data were collected using a structured, self-administered online questionnaire developed specifically for this study through a review of the relevant literature on community pharmacy practice and pharmacy workforce studies [13, 26–29]. It was administered in English only and therefore did not require translation. Prior to data collection, it was pilot-tested with 10 community pharmacists to assess clarity, comprehensibility, and feasibility. Minor amendments were made based on their feedback, and these pharmacists were not included in the final study sample.

The instrument comprised four sections: (1) demographic and workplace characteristics, including age, gender, years of experience, highest qualification, workplace setting, region, number of pharmacists per shift, opening hours per day, and average number of prescriptions dispensed per day; (2) availability and utilization of 26 professional pharmacy services, grouped into three domains: core pharmacy services, clinical and public health services, and advanced and access services; (3) eleven perceived barriers to service provision, rated on a five-point Likert scale (Strongly Disagree to Strongly Agree); and (4) overall satisfaction with the current scope of pharmacy services, rated from Very dissatisfied to Very satisfied. The complete questionnaire, including the exact wording of the 26 service items, is provided in Supplementary Material 1.

### Data collection procedure

Participants were recruited through a non-probability, convenience sampling approach via professional networks, pharmacy associations, and online distribution channels. The questionnaire was distributed electronically via Microsoft Forms over a period of 4 months. The survey link was shared through direct messaging and community pharmacy professional groups. Participants were provided with a brief introduction describing the study aims and were required to provide informed consent electronically before accessing the questionnaire.

### Outcome variable construction

Service provision was operationalized using two complementary summary measures: service availability and overall service utilization. All respondents were asked about the same 26 professional pharmacy services. For each service, respondents selected one of five response categories: Not available, Rarely (1–3 times/month), Sometimes (1–3 times/week), Often (≥4 times/week), or Always (daily). These categories were coded from 0 to 4, respectively. All respondents completed all 26 service items; therefore, there were no missing data for these items.

Service availability was calculated by recoding each service as 0 (Not available) or 1 (available at any frequency, from Rarely to Always) and summing across the 26 services, resulting in a score ranging from 0 to 26. This measure represents the breadth of professional pharmacy services reported as available by each respondent. Overall service utilization was calculated as the mean frequency score across the 26 services, resulting in a score ranging from 0 to 4. Given the heterogeneity of the services assessed, this measure was not intended to represent a single underlying latent construct but rather a pragmatic summary index of the overall frequency of service provision across the predefined set of services. It complements the availability score by capturing frequency of provision in addition to service breadth. Individual service frequencies were also reported separately to preserve differences across service types.

### Statistical analysis

All statistical analyses were performed using R (version 4.4.1; R Core Team, Vienna, Austria). The individual pharmacist was treated as the unit of analysis, with each respondent considered an independent observation. Categorical variables were summarized as frequencies and percentages; continuous variables were summarized as means and standard deviations (SD). Internal consistency of the survey domains was assessed using Cronbach’s alpha. Spearman rank-order correlation coefficients (r∼s∼) were used to assess associations between ordinal predictor variables and the two service provision outcomes: service availability score (number of services offered at any frequency, range 0–26) and service utilization score (mean frequency across all 26 services, range 0–4).

Seven candidate predictors were included in the multivariable logistic regression model based on their potential relevance to service utilization: prescription volume per day, years of experience, highest qualification, workplace setting, opening hours per day, number of pharmacists per shift, and age. All seven predictors were entered simultaneously irrespective of their bivariate statistical significance. Gender and region were excluded because of limited variability and sparse subgroup representation. Results are reported as adjusted odds ratios (aOR) with 95% confidence intervals (CI). For multivariable analysis, the utilization score was dichotomized at the sample median (1.27), with scores above the median classified as higher utilization and scores at or below the median as lower utilization. The cut-off was data-driven rather than prespecified and was used to obtain approximately balanced groups for binary logistic regression. Model performance was assessed using the likelihood-ratio test, Nagelkerke R^2^, and area under the receiver operating characteristic curve (AUC). Multicollinearity was assessed using variance inflation factors (VIFs). As a sensitivity analysis, multiple linear regression was additionally performed using the continuous utilization score as the outcome and the same seven primary predictors, to assess the robustness of findings independent of the median-based dichotomization. All statistical tests were two-tailed and a p-value of <0.05 was considered statistically significant.

### Ethical considerations

Ethical approval for this study was obtained from the Institutional Review Board of Taif University (IRB No. 47-109; approved on 11 December 2025). The study was conducted in accordance with the principles of the Declaration of Helsinki. Participation was voluntary, and electronic informed consent was obtained before participants commenced the questionnaire. No personally identifiable information was collected, and the anonymity of responses was maintained throughout data collection, analysis, and reporting.

## Results

### Participant characteristics

A total of 188 community pharmacy practitioners completed the survey (Table 1). The majority were male (n = 170, 90.4%), with a mean age of 31.3 years (SD 7.4; range 23–58). Most respondents held a PharmD degree (n = 118, 62.8%), followed by BPharm (n = 62, 33.0%). Years of experience were distributed across all categories: 40 respondents (21.3%) had less than 1 year of experience, 52 (27.7%) had 1–3 years, 37 (19.7%) had 4–6 years, 17 (9.0%) had 7–10 years, and 42 (22.3%) had more than 10 years. The majority of respondents worked in urban settings (n = 146, 77.7%), and most were based in Makkah region (n = 141, 75.0%). Regarding workplace characteristics, 90 respondents (47.9%) reported working with one pharmacist per shift and 85 (45.2%) with two pharmacists per shift, while 104 (55.3%) reported pharmacy operating hours of 17–23 h per day. Most respondents reported dispensing fewer than 50 prescriptions per day (n = 113, 60.1%), whereas 75 (39.9%) reported dispensing 50 or more prescriptions daily.

**TABLE 1:** Participant and pharmacy characteristics (n = 188).

Characteristic	n	%
Gender		
Male	170	90.4%
Female	18	9.6%
Age (years)	*Mean 31.3 (SD 7.4); range 23–58*	
Years of experience		
<1 year	40	21.3%
1–3 years	52	27.7%
4–6 years	37	19.7%
7–10 years	17	9.0%
>10 years	42	22.3%
Highest qualification		
BPharm	62	33.0%
PharmD	118	62.8%
Postgraduate (MSc/PhD)	8	4.3%
Workplace setting		
Urban	146	77.7%
Semi-urban	32	17.0%
Rural	10	5.3%
Region		
Makkah	141	75.0%
Riyadh	30	16.0%
Asir	10	5.3%
Other regions	7	3.7%
Pharmacists per shift		
1 pharmacist	90	47.9%
2 pharmacists	85	45.2%
≥3 pharmacists	13	6.9%
Opening hours/day		
<12 h	27	14.4%
12–16 h	25	13.3%
17–23 h	104	55.3%
24 h	32	17.0%
Prescriptions dispensed/day		
<50	113	60.1%
50–99	45	23.9%
100–199	23	12.2%
≥200	7	3.7%

### Availability and utilization of community pharmacy services

#### Core pharmacy services

Core pharmacy services demonstrated the highest rates of availability among respondents (Figure 1). Dispensing of prescription medications was reported as available by 97.9% of respondents, with a combined high-frequency utilization rate of 54.3%. Similarly, medication counselling was reported as available by 97.3% of respondents, with 42.0% reporting daily delivery and 14.9% reporting frequent delivery (combined: 56.9%). Management of minor ailments was reported as available by 80.9% of respondents; however, only 30.3% reported providing this service daily. Digital health services within this category showed markedly lower availability. Wasfaty electronic prescription processing was reported as available by 61.7% of respondents, while private insurance e-claims processing showed the lowest availability in this category, with 45.7% reporting that this service was unavailable.

**FIGURE 1:**
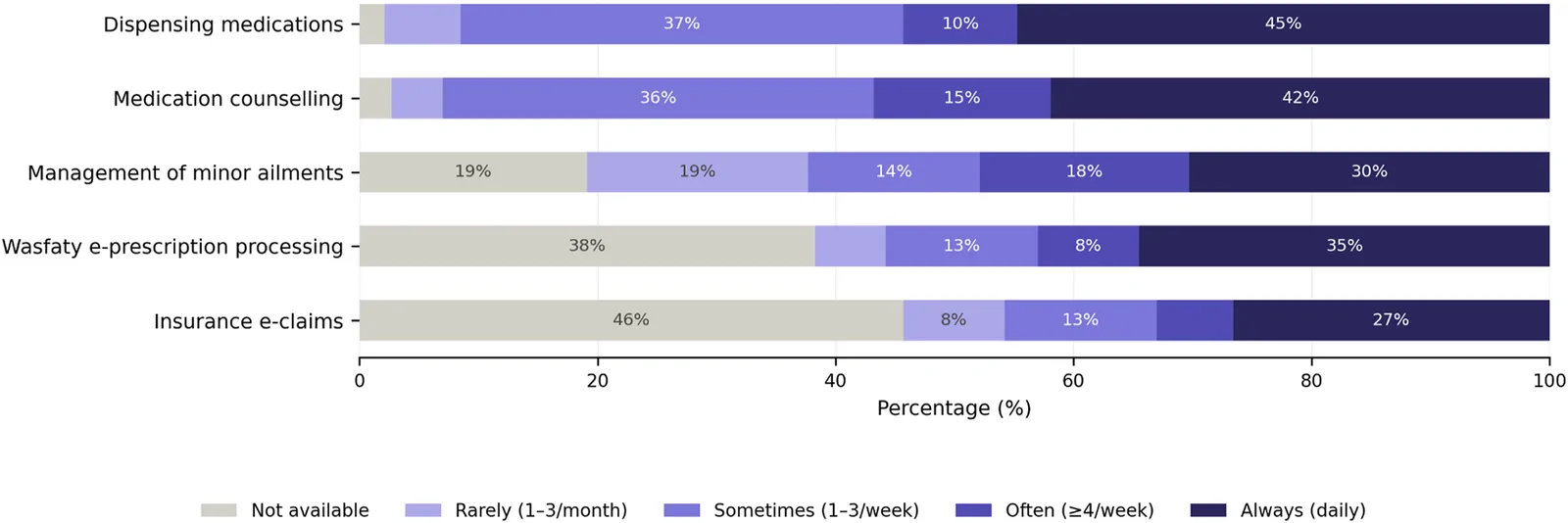
Core pharmacy services—availability and utilization frequency.

#### Clinical and public health services

Clinical and public health services exhibited a pronounced gradient of availability among respondents (Figure 2). Support for chronic disease management was reported as available by 88.3% of respondents, with 26.1% reporting providing this service rarely. Structured medication review was reported as available by 80.3% of respondents, though high-frequency utilization (often or always) was reported by only 30.9%. Lifestyle and behavioral counselling services were also reported as available by substantial proportions of respondents: weight management counselling (73.9%), emergency contraception counselling (68.6%), travel advice (66.5%), and smoking cessation counselling (66.0%). In each case, the most common utilization category was rare (1–3 times per month), reported by 29.3%, 29.3%, 37.2%, and 39.9% of respondents, respectively. Health screening services showed substantially lower availability. Weight screening was reported as available by 61.2% of respondents, while blood glucose and blood pressure screening were reported as available by only 44.1% and 42.0%, respectively. Vaccination was the least available clinical service in this category, with 80.9% of respondents reporting that this service was unavailable.

**FIGURE 2:**
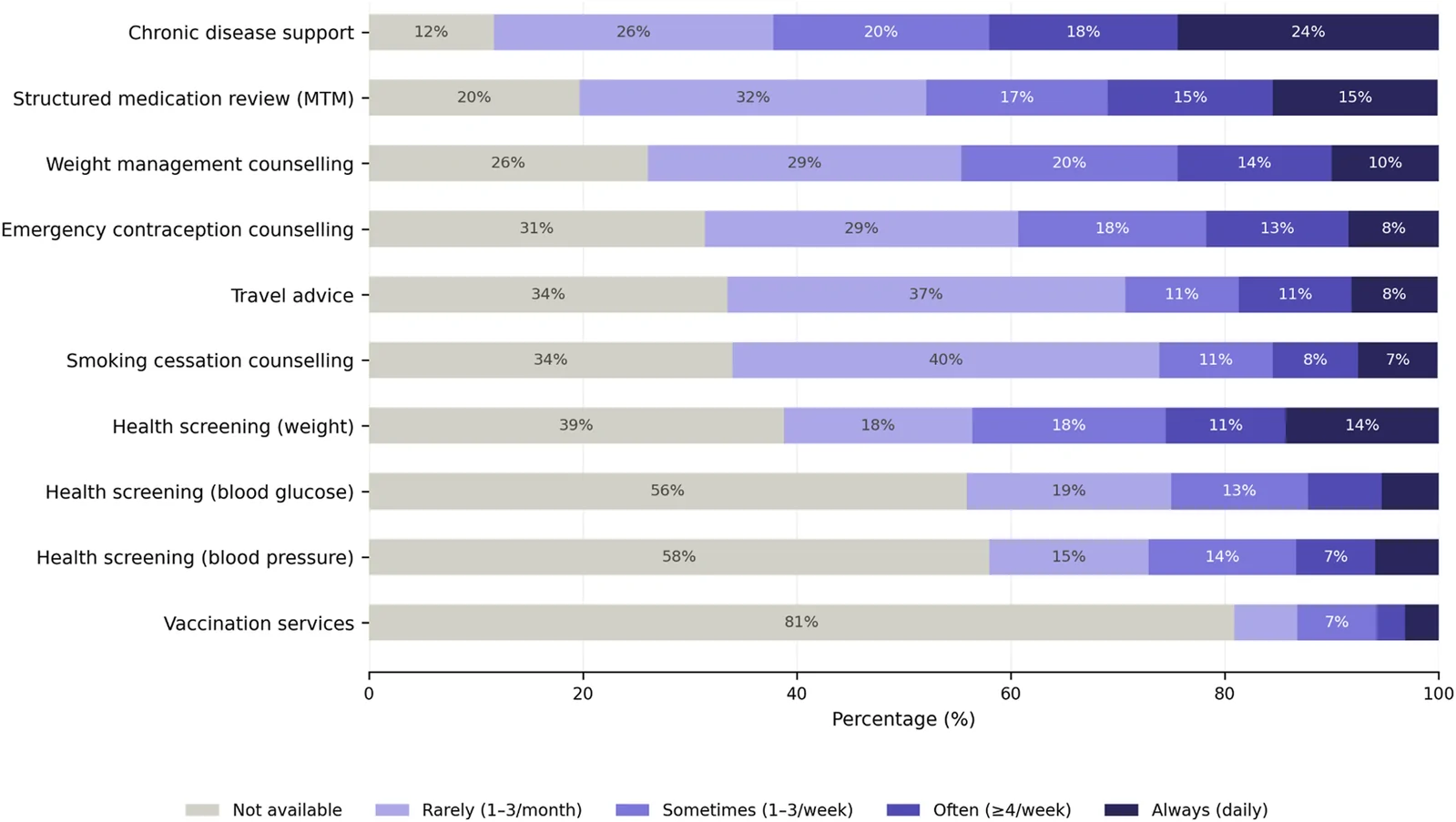
Clinical and public health services—availability and utilization frequency.

#### Advanced and access services

Advanced and access services were the least consistently available across the sample, with many services reported as unavailable by the majority of respondents (Figure 3). Home delivery was the most prevalent service in this category, reported as available by 72.9% of respondents, though daily provision was reported by only 17.6%. Digital adherence monitoring was reported as available by 53.2% of respondents, with 24.5% reporting frequent or daily provision. Pharmacovigilance and adverse drug reaction (ADR) reporting was available to 51.6% of respondents, yet high-frequency reporting was uncommon (12.8% often or always). Compounding services were reported as available by fewer than half of respondents (48.4%) and, where available, were predominantly provided on a rare basis (27.7%). Infrastructure-dependent services showed the lowest availability. Dosette or blister pack preparation was reported as unavailable by 65.4% of respondents, EHR access by 66.5%, and drive-thru dispensing by 72.3%. Private consultation rooms were reported as unavailable by 75.0% of respondents, with only 4.8% reporting daily use.

**FIGURE 3:**
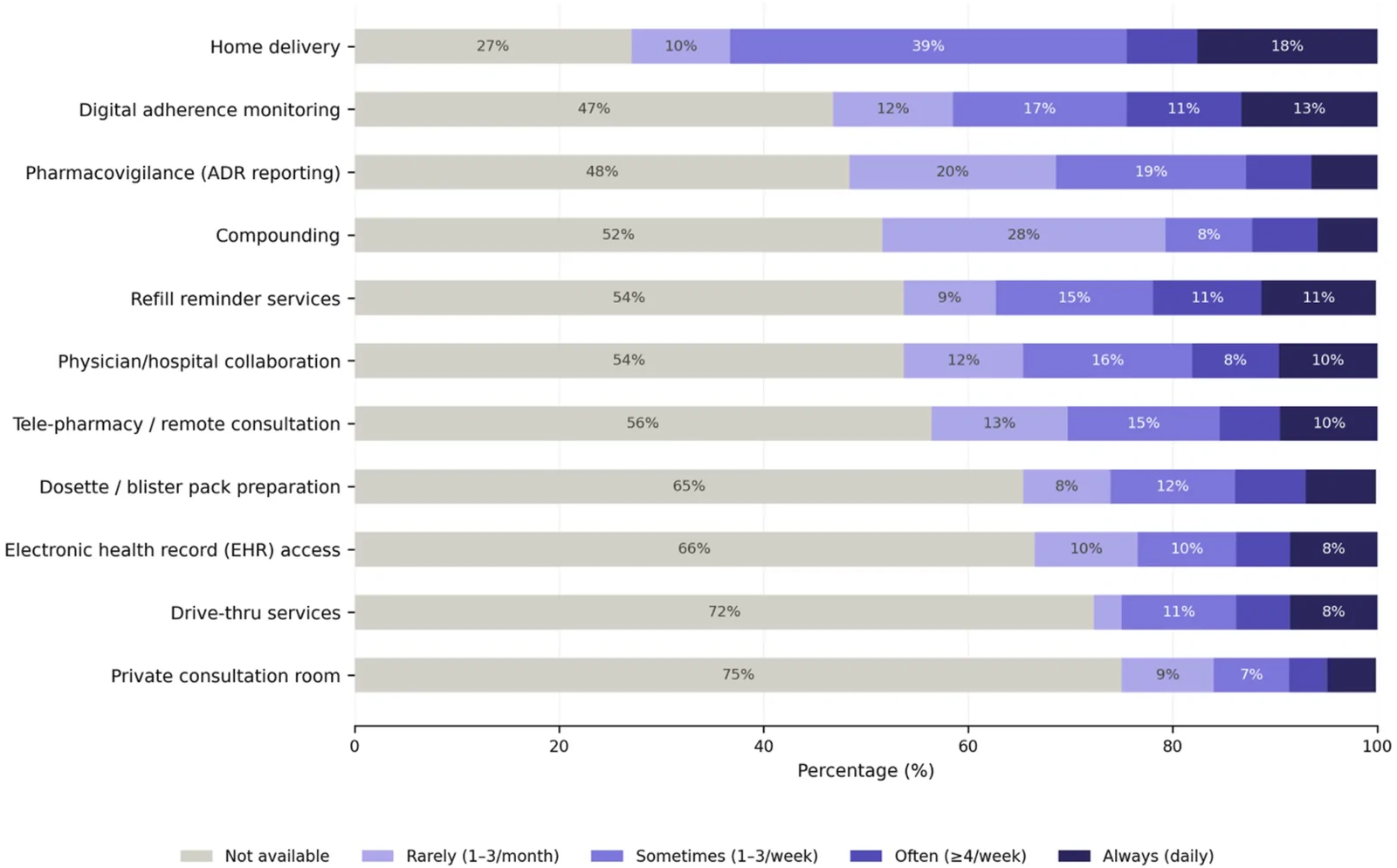
Advanced and access services—availability and utilization frequency.

### Factors associated with service provision

Spearman correlation analysis identified several factors significantly associated with both the availability and utilization of pharmacy services (Table 2). Prescription volume per day demonstrated the strongest positive correlation with service provision outcomes, showing moderate positive associations with both service availability (rs = 0.378, p < 0.001) and service utilization (rs = 0.430, p < 0.001). Similarly, years of professional experience were positively correlated with service availability (rs = 0.255, p < 0.001) and utilization (rs = 0.356, p < 0.001). Highest qualification was negatively correlated with both service availability (rs = −0.150, p = 0.040) and utilization (rs = −0.198, p = 0.006). In contrast, opening hours per day, workplace setting, and the number of pharmacists per shift were not significantly associated with either service availability or utilization (all p > 0.05).

**TABLE 2:** Spearman correlations between predictor variables and pharmacy service provision outcomes (n = 188).

Predictor variable	rs (availability)	p-value	rs (utilization)	p-value
Prescription volume/day	+0.378	<0.001***	+0.430	<0.001***
Years of experience	+0.255	<0.001***	+0.356	<0.001***
Highest qualification	−0.150	0.040*	−0.198	0.006**
Opening hours/day	+0.065	0.376 ns	+0.111	0.129
Workplace setting	−0.043	0.561 ns	−0.117	0.109
Pharmacists per shift	−0.014	0.854 ns	−0.063	0.392

rs = Spearman rank correlation coefficient; availability = number of distinct services provided at any frequency (range 0–26); utilization = mean service utilization frequency score (0–4 scale, averaged across 26 services). ***p < 0.001, **p < 0.01, *p < 0.05.

### Multivariate analysis of factors associated with high service utilization

The overall utilization score ranged from 0 to 4, with a median of 1.27. Using the median-based classification, 91 respondents (48.4%) were classified as having higher utilization and 97 (51.6%) as having lower utilization. In the fully adjusted multivariable model (Table 3), higher prescription volume was significantly associated with higher service utilization, with each category increase associated with 3.52-fold higher odds of higher utilization (aOR = 3.52, 95% CI: 2.15–5.75, p < 0.001). Years of experience was also positively associated with utilization (aOR = 1.56 per category increase, 95% CI: 1.04–2.34, p = 0.034). Compared with BPharm respondents, PharmD respondents had lower odds of higher utilization (aOR = 0.29, 95% CI: 0.11–0.79, p = 0.016), while postgraduate qualification was not significantly associated with utilization. Increasing age was also associated with lower odds of higher utilization (aOR = 0.91 per year, 95% CI: 0.83–0.99, p = 0.021). Opening hours, workplace setting, and number of pharmacists per shift were not significantly associated with utilization. The overall model was statistically significant (LR χ^2^ [8] = 56.20, p < 0.001), with a Nagelkerke R^2^ of 0.345 and an AUC of 0.807. No concerning multicollinearity was identified (maximum VIF = 3.48).

**TABLE 3:** Multivariate binary logistic regression of Factors Associated with High Service Utilization.

Variable	aOR	95% CI	p-value
Prescription volume per day, per category increase	3.52	2.15–5.75	<0.001
Years of experience, per category increase	1.56	1.04–2.34	0.034
PharmD vs. BPharm	0.29	0.11–0.79	0.016
Postgraduate (MSc/PhD) vs. BPharm	2.78	0.41–18.77	0.293 ns
Opening hours per day, per category increase	1.21	0.83–1.79	0.326 ns
Urban vs. Rural/Semi-urban	1.17	0.51–2.66	0.708 ns
≥2 pharmacists vs. 1 pharmacist	0.51	0.24–1.07	0.076 ns
Age, per year increase	0.91	0.83–0.99	0.021

aOR, adjusted odds ratio; CI, confidence interval; *ns*, not significant. All seven predictors were entered simultaneously. Ordinal categories were coded in ascending order as follows: prescription volume (<50, 50–99, 100–199, ≥200 prescriptions/day), years of experience (<1, 1–3, 4–6, 7–10, >10 years), and opening hours (<12, 12–16, 17–23, 24 h/day); the reported aOR, for each ordinal predictor represents the change in odds associated with a one-category increase. Reference categories: BPharm (qualification), rural/semi-urban (workplace setting), and 1 pharmacist per shift. a Postgraduate estimate is based on n = 8 respondents; the wide confidence interval reflects sparse data and this estimate should be interpreted with caution.

As a sensitivity analysis, multiple linear regression using the continuous utilization score and the same seven predictors produced findings consistent with the primary analysis (R^2^ = 0.260; adjusted R^2^ = 0.227). Prescription volume (β = 0.347, p < 0.001) and years of experience (β = 0.211, p = 0.002) remained significant positive predictors, while age was inversely associated with utilization (β = −0.029, p = 0.039). The postgraduate group was also positively associated with utilization (β = 0.667, p = 0.023), although this estimate should be interpreted cautiously because of the small subgroup size (n = 8). Other predictors were not significant.

### Internal consistency and correlations between survey domains

Internal consistency was acceptable to high across the survey domains, with Cronbach’s alpha values of 0.796 for core dispensing and traditional services, 0.895 for clinical and advanced patient care services, 0.843 for infrastructure and professional collaboration, and 0.839 for barriers to pharmacy service provision.

Core dispensing and traditional services were strongly positively correlated with clinical and advanced patient care services (rs = 0.836, p < 0.001) and moderately positively correlated with infrastructure and professional collaboration (rs = 0.670, p < 0.001). Barriers were not significantly correlated with core dispensing and traditional services (rs = 0.105, p = 0.151) or clinical and advanced patient care services (rs = 0.064, p = 0.381). A weak positive correlation was observed between barriers and infrastructure and professional collaboration (rs = 0.155, p = 0.034.

### Perceived barriers to service provision

Respondents identified several barriers that may hinder the provision and utilization of pharmacy services (Figure 4). The most frequently reported barriers were lack of clear regulations (55%), high workload and time constraints (53%), and Wasfaty-related administrative burden (51%). More than one-third of respondents also agreed that limited training or continuing professional development (39%), technical or software issues (36%), and lack of financial incentives (36%) negatively affected service provision. In contrast, medication stock shortages (28%), high competition between pharmacies (26%), and insurance restrictions (24%) were less commonly perceived as barriers, with a greater proportion of respondents disagreeing than agreeing with these statements.

**FIGURE 4:**
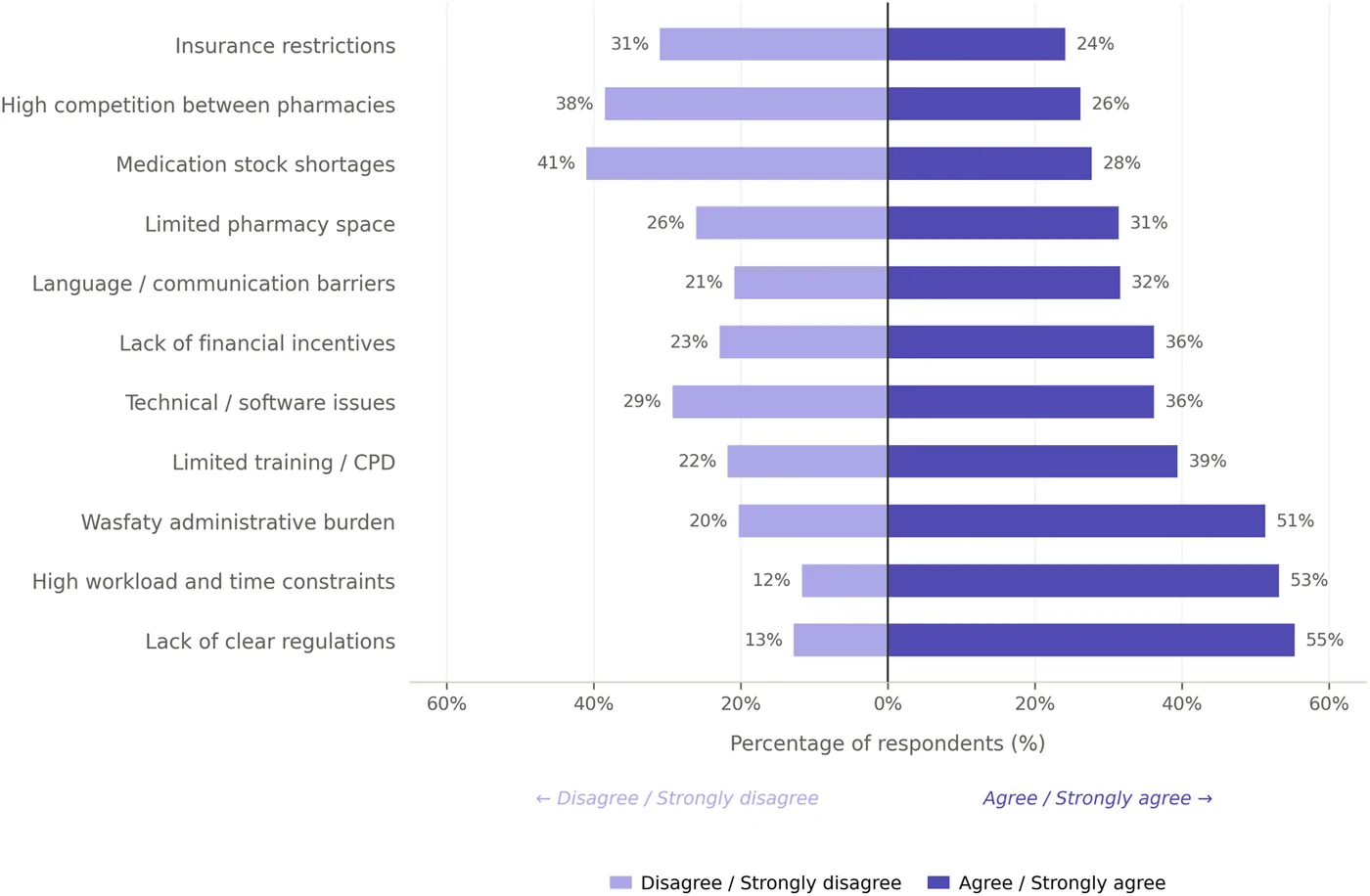
Perceived barriers to community pharmacy service provision.

### Overall satisfaction with current service scope

Overall satisfaction with the current scope of pharmacy services was rated on a five-point scale. As shown in Figure 5, most respondents reported neutral satisfaction (n = 80, 42.6%), followed by satisfied (n = 63, 33.5%), dissatisfied (n = 17, 9.0%), very satisfied (n = 16, 8.5%), and very dissatisfied (n = 12, 6.4%). Taken together, only 42.0% of respondents expressed positive satisfaction (satisfied or very satisfied), while 15.4% expressed dissatisfaction.

**FIGURE 5:**
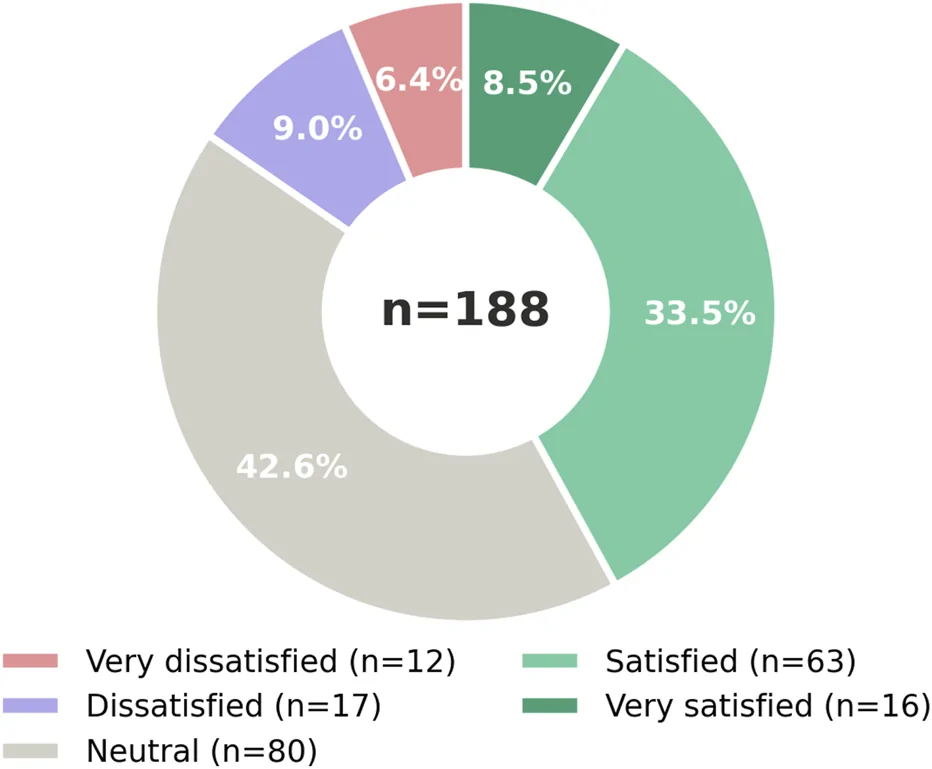
Overall satisfaction with the current scope of pharmacy services (n = 188). Inner value indicates total sample size.

## Discussion

This study provides an assessment of professional pharmacy services among community pharmacists in selected regions of Saudi Arabia. Core pharmacy services, particularly medication dispensing and counselling, were commonly reported, whereas the availability and routine utilization of clinical, preventive, and advanced services were more variable. Service utilization was associated with selected professional and operational characteristics, particularly prescription volume, while regulatory, operational, and administrative challenges were frequently reported as barriers to service provision. These findings highlight opportunities to support the expansion of professional pharmacy services and strengthen the role of community pharmacists in patient-centered healthcare delivery.

The widespread availability of core pharmacy services observed in this study reflects the continued central role of community pharmacists in medication dispensing and patient counselling. Similar findings have been reported internationally [30, 31]. However, the findings also demonstrate that the transition toward expanded clinical and public health services remains incomplete. Although services such as chronic disease management, medication review, smoking cessation counselling, and lifestyle interventions were available in many pharmacies, they were often delivered infrequently. This discrepancy between service availability and actual utilization has also been reported in studies from Europe, Australia, and the Middle East, suggesting that introducing new services alone is insufficient to ensure their routine integration into pharmacy practice [30, 32, 33].

Preventive health services were among the least frequently available services. This finding contrasts with healthcare systems where community pharmacists have become well-established providers of immunization and preventive care [34]. In Saudi Arabia, although national healthcare reforms have expanded pharmacists’ professional responsibilities, implementation appears to remain heterogeneous across community pharmacies. These findings suggest an opportunity to further integrate community pharmacists into primary healthcare and expand access to preventive and pharmaceutical care services [14]. Advanced pharmacy services requiring dedicated infrastructure or digital integration were also less commonly available. Limited access to electronic health records, private consultation rooms, adherence monitoring systems, and pharmacovigilance activities was frequently reported. These infrastructure limitations may contribute to the lower provision of advanced services, although this relationship was not directly evaluated in the present study. Similar barriers have been described in previous pharmacy practice studies and emphasize that service expansion depends not only on pharmacists’ clinical competence but also on the availability of appropriate infrastructure and technological support [13, 35–37].

One of the most notable findings of this study was the strong association between prescription volume and service utilization. Respondents reporting higher prescription volumes had higher service utilization, even after adjustment for other measured variables. Possible explanations include differences in organizational capacity, staffing, patient contact, or operational resources, although these factors were not directly assessed in this study. International evidence similarly suggests that the provision of advanced pharmacy services is associated with dispensing volume and organizational characteristics, including pharmacy ownership and opening hours [38]. Observational workflow data also indicate associations between prescription volume, pharmacist staffing, and professional service activities [39].

Pharmacists consistently identified regulatory uncertainty, workload, and administrative responsibilities as the principal barriers to service provision. These findings align closely with previous international research, which has consistently identified insufficient regulatory support, inadequate staffing, time constraints, lack of reimbursement, and workflow pressures as major obstacles to implementing expanded pharmacy services [39, 40]. Together, these findings indicate that successful expansion of community pharmacy practice requires supportive policies with clear role definitions, sustainable funding mechanisms, adequate staffing and workflow redesign, and stronger organizational support, in addition to pharmacist education and training.

The findings have several implications for healthcare policy and community pharmacy practice in Saudi Arabia. First, national efforts should focus on establishing clear standards and regulatory frameworks that define and support professional pharmacy services beyond traditional dispensing. Second, reimbursement models for extended pharmacy services should be considered to encourage the routine delivery of medication reviews, chronic disease management, preventive health services, and patient counselling. Third, reducing administrative burden associated with digital prescribing systems and improving workflow efficiency may allow pharmacists to dedicate more time to direct patient care. Finally, investment in infrastructure, including consultation rooms, digital health integration, and continuing professional development, will be essential to support the evolving role of community pharmacists within the Saudi healthcare system.

This study has several strengths. It represents one of the first assessments of professional pharmacy services in some regions of Saudi Arabia community pharmacies using a multidimensional framework that simultaneously evaluated service availability, utilization, associated factors, perceived barriers, and pharmacist satisfaction. The inclusion of a broad range of professional services provides a more complete understanding of current pharmacy practice than studies focusing on individual services alone. However, several limitations should be acknowledged. The cross-sectional design precludes causal inference, while convenience sampling and the geographical concentration of respondents may have introduced selection and participation bias and limit generalizability. Because the number of pharmacists who received or viewed the survey invitation was unknown, a response rate could not be calculated, and potential non-response bias cannot be excluded. Self-reported service provision may be subject to recall and social desirability bias, and the study questionnaire, although pilot-tested with acceptable internal consistency, was not previously validated. Pharmacy-level identifiers were not collected; therefore, potential clustering within pharmacies could not be accounted for, which may have affected the precision of the estimates. The median-based dichotomization of utilization may have resulted in information loss, although findings were generally consistent in the continuous-outcome sensitivity analysis. Finally, sparse predictor categories resulted in imprecise estimates, and residual confounding from unmeasured factors cannot be excluded despite multivariable adjustment.

Future studies should evaluate pharmacy service provision using nationally representative sampling strategies and objective service utilization data obtained from pharmacy records. Longitudinal studies are needed to examine changes in community pharmacy practice as healthcare reforms continue under Saudi Vision 2030. Qualitative research involving pharmacists, patients, pharmacy owners, and policymakers would also provide deeper insight into barriers and facilitators affecting service implementation. Finally, intervention studies evaluating strategies such as reimbursement models, workforce expansion, digital integration, and targeted professional training could identify effective approaches for increasing the delivery of clinical and public health pharmacy services.

Overall, community pharmacists in selected regions of Saudi Arabia reported high availability of core pharmaceutical services, whereas clinical, preventive, and advanced services were less consistently available. Service utilization was associated with selected professional and operational characteristics, while regulatory, operational, and administrative barriers were also reported. Addressing these barriers, supporting workforce and infrastructure development, and strengthening the integration of community pharmacists into primary healthcare may help expand professional pharmacy services and enhance their contribution to healthcare delivery in Saudi Arabia.

## Data Availability

The raw data supporting the conclusions of this article will be made available by the authors, without undue reservation.
