# Supplementary material for: Availability, utilization, and factors associated with professional pharmacy services in community pharmacies in Saudi Arabia: a cross-sectional study
Source: J Pharm Pharm Sci. 2026 Sep 22;29:17413. doi: 10.3389/jpps.2026.17413 (PMC13639138; doi:10.3389/jpps.2026.17413)
Supplement: Supplementary file 1 [file DataSheet1.pdf]

Availability and Utilization of Community Pharmacy Services and Associated Barriers in Saudi Arabia

We are a research group from the College of Pharmacy, Taif University, conducting a cross-sectional study to assess the *availability and utilization* of community pharmacy services in Saudi Arabia. Your participation in this survey will contribute valuable insights into the current and emerging roles of community pharmacists in patient care and public health.

The survey should take no longer than 5 minutes to complete. All responses will remain anonymous and strictly confidential. Only the research team will have access to the collected data, which will be used solely for academic and research purposes. Participation is voluntary, and you may withdraw from the survey at any time without providing a reason.

Target Group: Licensed pharmacists or supervised pharmacy interns currently working in community pharmacies in Saudi Arabia.

If you have any questions, please contact the principal investigators:

- Dr. Wael Y. Khawagi – w.khawagi@tu.edu.sa
- Dr. Abdullah A. Alshehri – a.aalshehri@tu.edu.sa

Consent to Participate

I have read and understood the information provided above about this study. I understand that my participation is voluntary and that all information I provide will remain confidential and used solely for research purposes.

☐ **I agree to participate in this study.**

Section A – Demographic Information and Workplace Information

1. Role (Pharmacist / Manager / Intern)
2. Years of experience in community pharmacy practice (<1 / 1–3 / 4–6 / 7–10 / >10)
3. Highest qualification (BPharm / PharmD / MSc / PhD / Other)
4. Gender (Male / Female)
5. Age: _________________________
6. Workplace setting — Urban, Semi-urban or Rural
7. Region (Riyadh / Makkah / Madinah / Eastern / Qassim / etc.)
8. Number of pharmacists per shift (1 / 2 / 3+)
9. Opening hours per day (<12h / 12–16h / 16–20h / 24h)
10. Average number of prescriptions dispensed per day (<50 / 50–99 / 100–199 / ≥200)

Section B – Services Currently Offered

Please indicate how frequently each of the following services is provided in your pharmacy during the last 12 months:

	**Not available**	**Rarely** *(≤1 time/month)*	**Sometimes** *(1–3 times/week)*	**Often** *(≥4 times/week)*	**Always** *(daily)*
Dispensing prescription medications					
Counseling on medications					
Wasfaty e-prescription processing					
Insurance e-claims					
Management of minor ailments					
Support for chronic disease management					
Structured Medication review or Medication therapy management (MTM)					
Digital Adherence Monitoring					
Pharmacovigilance (ADR reporting)					
Health screening (Blood pressure)					
Health screening (Blood glucose)					
Health screening (weight)					
Vaccination services					
Smoking cessation counseling					
Weight management counseling					
Travel advice					
Emergency contraception counseling					
Home delivery					
Drive-thru					
Tele-pharmacy or remote video consultation					
Compounding					
Private consultation room					
Refill reminder services					
Collaboration with physicians or hospitals for shared care					
Preparation of Dosette boxes / Blister packs for medication organization					
Electronic Health Record (EHR) Access					

Section C – Challenges and Barriers

Please rate how much each of the following barriers affects the usage of pharmacy services:

	Strongly Disagree	Disagree	Neutral	Agree	Strongly Agree
Limited public awareness of pharmacy services					
High workload and time constraints					
Lack of financial incentives or reimbursement					
Lack of clear regulations or policies governing service delivery					
Limited training or continuing education opportunities					
Limited pharmacy space or infrastructure					
Technical or software-related issues					
Frequent medication stock shortages					
Language and communication barriers					
Insurance restrictions or limited coverage					
High competition between pharmacies					
Administrative burden associated with Wasfaty program					

Q: Overall, how satisfied are you with the current scope and range of professional services offered in your pharmacy?

- Very dissatisfied – The current services are highly limited and do not meet patient or professional needs.
- Dissatisfied – The services offered are below expectations or insufficient to provide optimal care.
- Neutral – The current services are adequate but could be improved.
- Satisfied – The services meet most expectations and patient needs.
- Very satisfied – The services are comprehensive, effective, and meet both professional and patient needs fully.

THANK YOU FOR COMPLETING THIS QUESTIONNAIRE
